# Some Medical Quacks

**Published:** 1881-02

**Authors:** 


### SOME MEDICAL QUACKS.

In spite of all moral condemnation, one cannot avoid a certain admiration for a bold and successful imposter. Boldness and

*"Advantages and Accidents of Artificial Anæsthesia," by Laurence Turnbull, M.D., Philadelphia, 1877. p. 128.

shrewdness are captivating in themselves. Many people like a trickster, if he is only bold and entirely without scruple. To every condemnation of his morals, men rejoin that he is "mighty smart," or, as they say in England of a famous living statesman, "awfully clever." But one cannot help being amused even with these imposters. A vulture is interesting from some standpoints.

The common resort of quacks in the times of a generation or more ago was Thompsonianism. I have heard that Thompson's little book, containing all the secrets of therapeutics, was sold for twenty dollars, the buyer binding himself not to communicate these mysteries to any other person. As the Thompsonians used only vegetable remedies, and for the most part simples, they were called "root-doctors," and from the use of "steam sweats," by means of boiled Indian corn packed about the patient, they got the *sobriquet* at the West of "corn-doctors," but more commonly of "steam-doctors." Any bold faced ignoramus might set up for a steam-doctor: it was Gil Blas's "universal dissolvent" come back again; for there is nothing new even in quackery. Some of the theories which the root-doctors came to hold were very amusing. I know a minister of prominence in the West, who was once a "student" or office-boy for one of them. He relates that the doctor sent him into the woods to get some of the inner bark of the butternut tree.

"Tom," said the doctor, as he departed, "I want you to scrape this bark downward. It is for a cathartic. Don't you scrape it upward, or it will be an emetic. And whatever you do, Thomas, don't you scrape it both ways. If you do, nobody on earth can tell how it will act."

The rarest specimen of the quack that I have ever known lived in an important city on the upper Mississippi, and practiced curing by mesmerism. It was better than a play to see grave clergymen, lawyers, and other prominent citizens, file into Dr. X's office of a morning, to have the solemn old humbug put his magnetic paw upon their heads.

Meeting "Doctor" X. one day in a public library, I sought to hear his theory of healing. He expounded it, almost in these words: "I put my hand upon the patient's head, and bring the sensorium of my brain into contact with the sensorium of the patient's brain. Then I send a subtle current of etherium all over the patient's system, stimulating all his organs into activity. Then I make my examination. I do not want the patient to tell me anything about his symptoms -- symptoms are apt to mislead -- but I begin with the upper lobe of the brain; if I find that all right I proceed to the middle lobe; then the lower lobe, or cerebellum; and if I find a coagulation of blood at the top of the spinal cord, I know that the patient has epilepsy, and so on."

A Jew, by the name of Quohn, was my neighbor. He was a merry-hearted fellow, in spite of the intolerable agony of eighteen years of asthma, which a little later caused his death. He went to see Doctor X., of course, and the exertion of climbing the doctor's steps set him a wheezing like the steam engine at a blast furnace. Placing his hand on Mr. Q.'s head, the wise doctor pronounced the patient to be suffering from asthma. This was a remarkable token of skill, and the patient suffered himself to come under the doctor's hand for five minutes a day during the next five or six weeks, at fifty cents each time. At last, finding his asthma growing steadily worse, he gave over, laughing merrily at his own stupidity.

"I t'ink," he said to me one day, "t'at Doctor X. has cot a coot deal of magnetic power."

"What makes you think so?" I asked.

"How could he traw eighteen dollars and a half out of my pocket if he hadn't?" he gasped.

I have heard, or read, that there was in one of the larger western towns a man who called himself an "Indian doctor," who was all the vogue, to the great chagrin of the regular physicians. At last he had an amputation to perform, and the consulting physicians, regardless of the patient, stood off to see the ignorant man make a fool of himself. To their surprise, he performed the operation well. One of the doctors took him aside and inquired how he knew so much of surgery; upon which the quack showed a diploma, saying that he knew he should starve if he did not pretend to quackery. Upon this being told to the others, one of them said, "We'll ruin him now," which they did by reporting everywhere that he was a regularly educated physician. One of the rarest quacks I have ever known was a man whose mind was positively feeble in everything but cunning. He was always boasting of his success. He took an active part in politics and secret societies, for the sake of talking about his patients, until he became a byword. Once in a political meeting he was appointed on a committee. Instantly he was on his feet.

"Mr. Chairman," he drawled, "I hope you'll excuse me. I must leave the house at once to see a patient."

"Mr. Chairman," cried another, "I hope you'll excuse Doctor W., and let him go to see this patient. This is the first patient he has had in a month."

Some years ago, a fellow lay dead drunk in one of the streets of New York. Some rollicking medical student pushed through the crowd that surrounded the drunken man, and declared immediately that the man was not drunk, but was suffering from a bad attack of strabismus, and was likely to die. This horrified the crowd, and each man repeated the story to his neighbor, with every pretense of knowing all about the dreadful disease. At last one of the medical professors came to the outskirts of the crowd and made inquiry, upon which he learned that there was a man dying of strabismus.

Just this trick of imposing on the imagination by words not understood, the makers of medicine almanacs play from year to year. And not they alone. How many physicians, who should know better, do the same thing, by affecting a learned jargon quite incomprehensible to common folks. And how many have made reputations to which they are not entitled, by adroitly pretending that their cases were very bad ones.

As the world comes out of its babyhood, and men understand more and more how inflexible are the laws of life, the quack and miracle-monger will find their occupation gone. Nothing is so much needed as a good, healthy skepticism.—*Edward Eggleston, in Scribner's Monthly.*

